# Broken Heart Syndrome Secondary To Liver Abscess

**DOI:** 10.7759/cureus.6804

**Published:** 2020-01-28

**Authors:** Raunak Nair, Hassan Lak, Taha Ahmed, Anjli Maroo

**Affiliations:** 1 Internal Medicine, Cleveland Clinic - Fairview Hospital, Cleveland, USA; 2 Internal Medicine, Cleveland Clinic Foundation, Cleveland, USA; 3 Cardiology, Cleveland Clinic - Fairview Hospital, Cleveland, USA

**Keywords:** broken heart syndrome, liver abscess, sepsis, takotsubo, acute coronary syndrome

## Abstract

Takotsubo cardiomyopathy is a well-known mimicker of acute coronary syndrome and is most often seen in postmenopausal women. Though it is most commonly observed after a stressful emotional episode, several infections have also been shown to precipitate this. Here, we describe a unique case of takotsubo cardiomyopathy that was precipitated by liver abscess induced sepsis.

## Introduction

Takotsubo cardiomyopathy (TTCM) also known as “broken heart syndrome” is transient left ventricular dysfunction most commonly associated with emotional or physical stress. It can often mimic an acute myocardial infarction and is characterized by the absence of obstructive coronary artery disease or plaque rupture on angiography [1]. Although the exact mechanism contributing to this condition remains undescribed, catecholamine surge has been shown to have a role in precipitating TTCM [2-3]. Several infections have also been shown to precipitate TTCM; however, there have been very few cases describing its association with liver abscess [4-7]. We present a rare case of Takotsubo cardiomyopathy precipitated by the liver abscess.

## Case presentation

**Figure 1:**
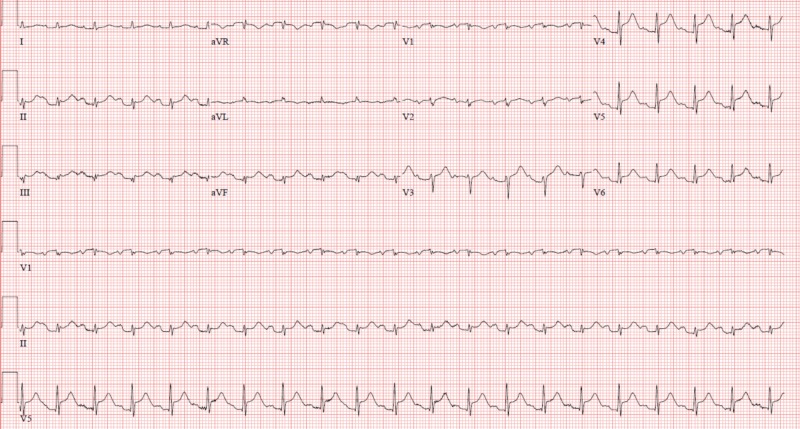
Electrocardiogram on presentation showing diffuse ST elevation.

An 85-year-old lady with a past medical history significant for hypertension and breast cancer (status post-mastectomy and radiation), presented to our hospital with complaints of generalized weakness and fever for 2 days. Apart from a productive cough and rhinorrhea, she denied any other associated symptoms. Systemic examination did not reveal any significant findings. Her vitals on presentation revealed that she was tachycardic (HR-98), normotensive (133/63), and afebrile. Initial labs were significant for leukocytosis (17.84), hypokalemia (2.9), elevated pro-brain natriuretic peptide (BNP) (4415), and normal lactate (1.4). Troponin T and creatine kinase-MB (CK-MB) were elevated and were trending up (Trop T - 0.289 to 0.896). Electrocardiogram (EKG) showed diffuse ST-segment elevations in the anterior and inferior leads (Figure 1).

**Figure 2:**
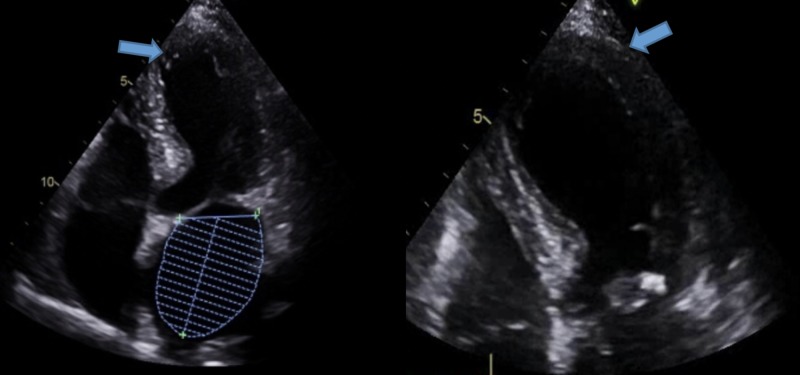
Apical four-chamber view showing apical hypokinesia and ballooning characteristic of Takotsubo cardiomyopathy. The image on the right is an expanded view of the image on the left. Both images here show the characteristic LV ballooning of the apex.

**Figure 3:**
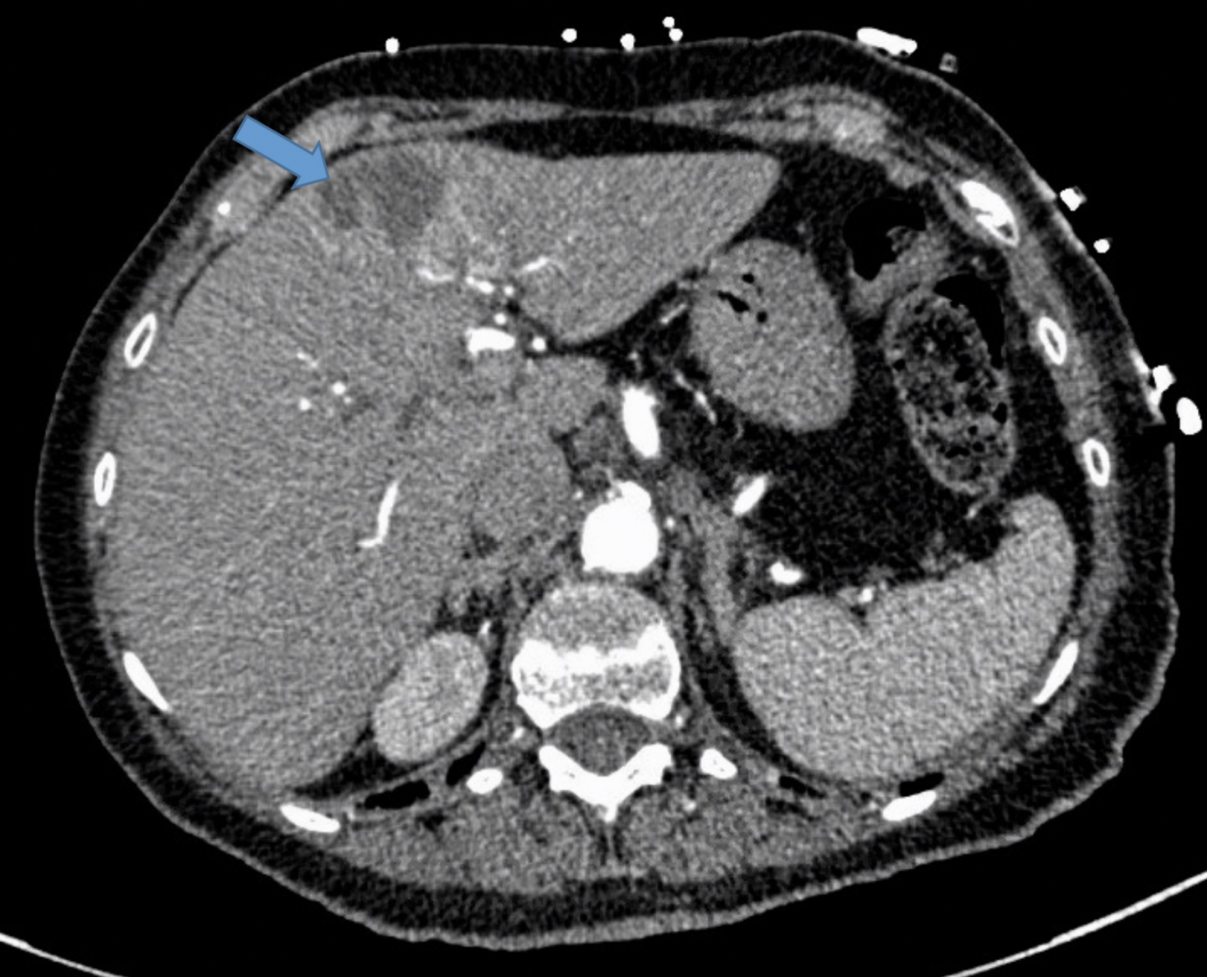
Computed tomography scan of the abdomen revealing 2 x 3 cm hypodensity in the left lobe of the liver.

The chest X-ray was unremarkable. A computed tomography (CT) scan of the chest revealed hilar lymphadenopathy, right lower lobe nodule, and a hypodensity in the left lobe of the liver. A bedside echocardiogram revealed trivial pericardial effusion with no wall motion abnormalities. She was diagnosed as having pericarditis and was started on 81 mg aspirin. Soon after, the patient went into shock and had to be started on vasopressors. Blood and urine cultures were also sent and the patient was started on broad-spectrum antibiotics. An urgent echocardiogram showed apical hypokinesia suggestive of TTCM with an ejection fraction (EF) of 40% (Figure 2). A left heart catheterization was also done, which revealed non-obstructive coronary artery disease. All cultures remained negative. To further explore the cause of the shock, a CT scan of the abdomen was done, which showed a 2 x 3 cm hypodense mass in the left lobe of the liver (Figure 3).

**Figure 4:**
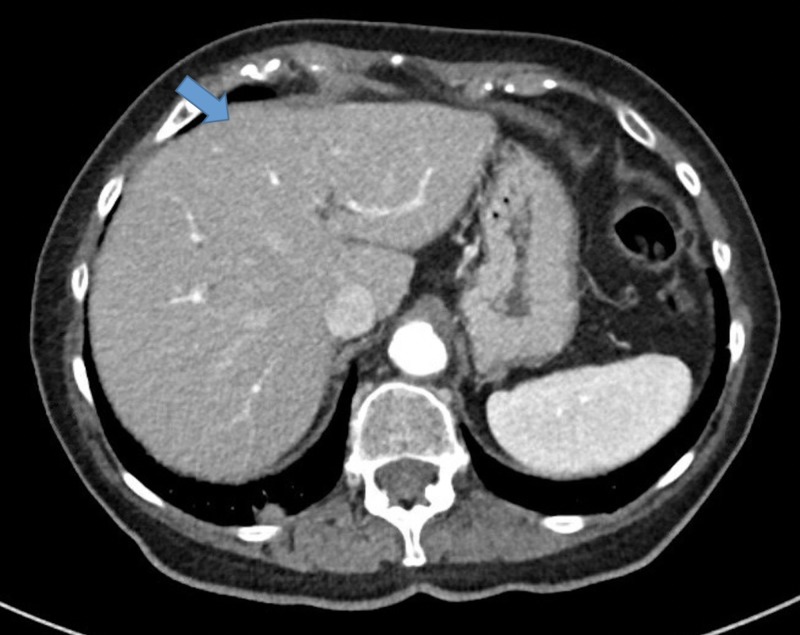
Computed tomography scan of the abdomen after antibiotics showing resolution of the hypodensity in the liver.

**Figure 5:**
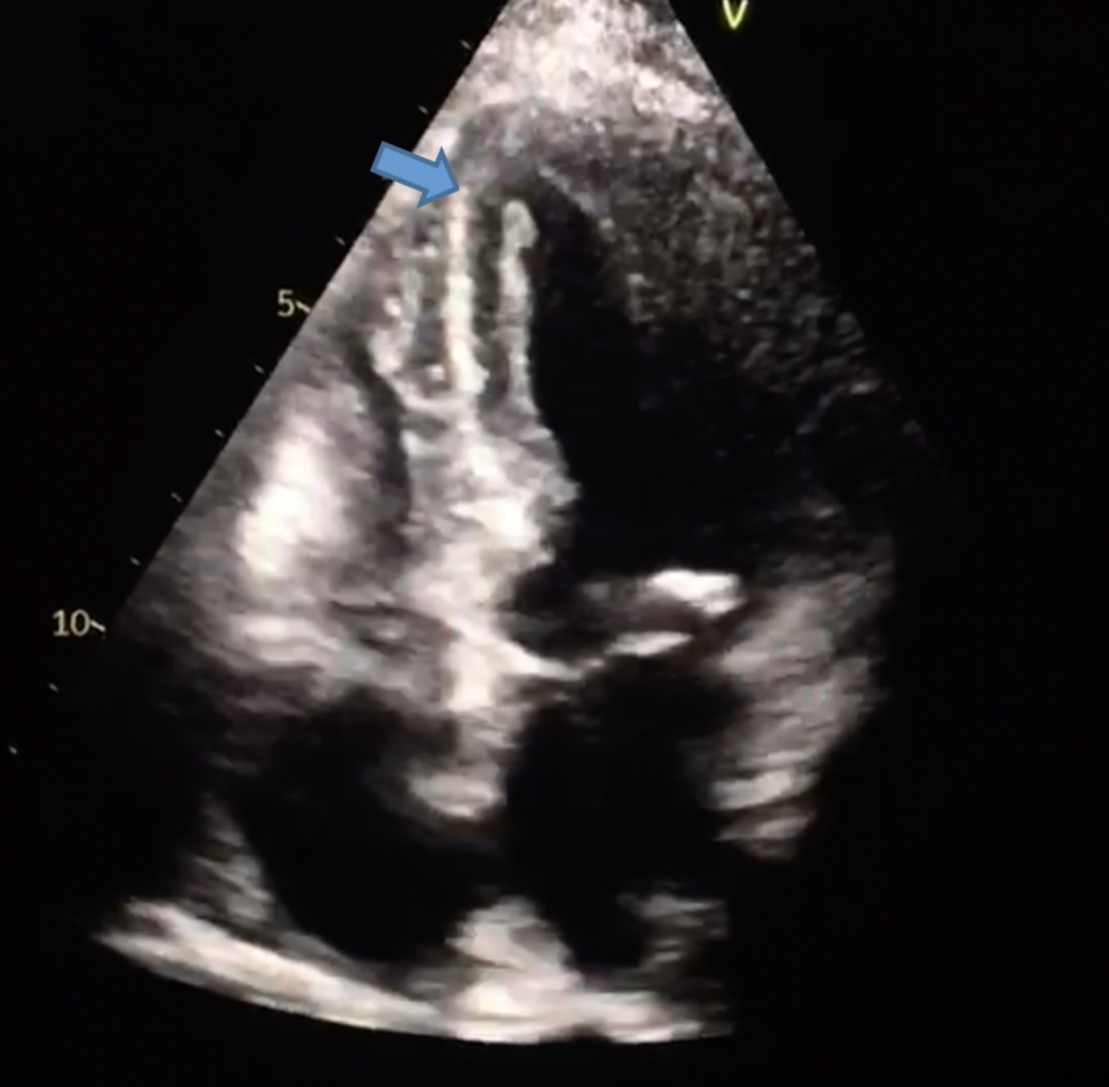
Apical four-chamber view in the echocardiogram repeated after 8 weeks showing normal left ventricular contraction.

The patient underwent a biopsy of the mass, which showed features suggestive of hepatic abscess. She was continued on antibiotics and she gradually improved. The patient was eventually discharged on 6 weeks of intravenous antibiotics. A CT scan of the abdomen was repeated in 8 weeks, which showed resolution of the hepatic abscess (Figure 4). An echocardiogram was also repeated around this time, which showed complete resolution of prior cardiomyopathy, an EF of 60%, and no residual wall motion abnormalities (Figure 5).

 

## Discussion

TTCM is a well-known mimicker of an acute coronary syndrome (ACS) and contributes to around 2% of all patients admitted with suspicion of ACS [8]. It is characterized by apical ballooning and transient left ventricular dysfunction, which usually improves within a few days or weeks. Although this condition is most commonly seen in postmenopausal women typically after an emotional stressor, it has also been reported in younger individuals, men, and even children [4,9].

The exact mechanism leading to TTCM remains unknown. However, catecholamine-induced cardiotoxicity, microvascular dysfunction, stimulus trafficking, coronary vasospasm, and estrogen deficiency are the most prominent theories described behind its occurrence [10-11]. Several studies have shown that serum catecholamine levels are elevated during the acute phase of TTCM [2-3]. Wittstein et al. also showed that in patients with TTCM, circulating catecholamine levels were several times higher than that in patients with myocardial infarction [2]. Such supraphysiological levels can cause myocardial damage and cardiac dysfunction. Since infections and all stressful events lead to increased catecholamine surge, they can precipitate TTCM via this mechanism. It is also believed that high levels of circulating epinephrine could initiate a change in intracellular signal trafficking from Gs (Stimulatory) to Gi (inhibitory) [12]. The net effect of this would lead to negative inotropy, and since the apex has the maximum density of beta-adrenoreceptors, it was predominantly affected, leading to the characteristic apical ballooning. The increased prevalence in postmenopausal women might also imply that estrogen deficiency has a role in precipitating TTCM. In a study by Cao et al., the administration of epinephrine in ovariectomized rats was seen to cause more cardiac dysfunction than that in rats with intact estrogen hormones, thus implying a potential cardioprotective role for estrogen [13].

TTCM can have a wide array of presentations. Patients can often present with chest pain, shortness of breath, nausea, vomiting, palpitations, or syncope. Around 30% of cases can also be asymptomatic [12]. They can also have EKG changes and troponin elevation similar to acute myocardial infarction. In fact, ST-segment elevation can be seen in up to 44% of cases of TTCM [13]. Most cases of TTCM are seen in 1-5 days following a stressor, which can be physical or emotional. Since TTCM can be a recurring event, effort should be made to identify the presence of any potential stressors. Diabetes mellitus, cannabis use, and underlying panic/anxiety disorder are well-known risk factors for TTCM. There are several accepted criteria for diagnosing TTCM [14-16]. However, the revised Mayo Clinic criteria are the most widely accepted one (Table 1). Although the suspicion for TTCM can be established during an acute presentation, the diagnosis often involves invasive testing (such as coronary angiography) to exclude an acute myocardial infarction due to an obstructive lesion. Cardiac magnetic resonance imaging (MRI) can also aid in the diagnosis of TTCM. The absence of macroscopic fibrosis as evidenced by the lack of delayed gadolinium enhancement on a cardiac MRI is characteristic of TTCM [17]. In our patient, the normal coronaries on angiography, coupled with the characteristic apical ballooning on echocardiogram aided us in our diagnosis.

**Table 1:** Revised Mayo Clinic criteria used to diagnose takotsubo cardiomyopathy.

Revised Mayo Clinic Criteria [14]
1. Transient hypokinesis, akinesis, or dyskinesis of the left ventricular midsegments with or without apical involvement; the regional wall motion abnormalities extend beyond a single epicardial vascular distribution; a stressful trigger is often, but not always present.
2. Absence of obstructive coronary disease or angiographic evidence of acute plaque rupture.
3. New electrocardiographic abnormalities (either ST-segment elevation and/or T-wave inversion) or modest elevation in cardiac troponin.
4. Absence of pheochromocytoma or myocarditis.

Although stress cardiomyopathy is a reversible condition, it can often lead to several complications before it resolves, which contributes to in-hospital mortality of around 5% [18]. Acute heart failure, cardiogenic shock, left ventricular outflow tract obstruction, arrhythmias, systemic thromboembolism, and intramural hemorrhage or rupture are few potential complications of TTCM, which contribute to the in-hospital mortality [15]. Prompt diagnosis and accurate management are key. However, there are no specific guidelines directing the management of patients with stress cardiomyopathy; supportive care and treatment of complications remain the cornerstone of therapy [19]. Since TTCM resembles an acute myocardial infarction on presentation, initial medical therapy involves administering antiplatelet agents, statins, and beta-blockers. Subsequent therapy is based on the clinical and hemodynamic status of the patient. Patients with heart failure should be managed with standard heart failure therapy. Venodilators, diuretics, and arteriodilators are often used. In patients with hemodynamic instability or shock, it is important to distinguish the presence or absence of left ventricular outflow tract obstruction (LVOT), which is seen in around 10-25% of patients. In the absence of LVOT, ionotropic agents such as dobutamine or dopamine can be used to augment left ventricular function. If moderate to severe LVOT is suspected on echocardiogram, inotropes should not be used as they can worsen the obstruction. Instead, beta-blockers and fluids should be used to augment the preload [20]. In patients with LV thrombus, anticoagulation is recommended for at least 3 months [15]. Studies have failed to show a decrease in the recurrence of TTCM with chronic use of beta-blockers and hence is not recommended [14].

## Conclusions

In summary, any event leading to the release of supraphysiological levels of catecholamines can predispose the person to develop TTCM. So, it is important to consider this condition in patients presenting elevated troponins and chest pain in the setting of infection. Although the association between several infections and TTCM has been described before, to our knowledge this is the first case describing the occurrence of TTCM secondary to a liver abscess related sepsis. 
